# Evaluation of Consistency in Spheroid Invasion Assays

**DOI:** 10.1038/srep28375

**Published:** 2016-06-23

**Authors:** Liliana R. Cisneros Castillo, Andrei-Dumitru Oancea, Christian Stüllein, Anne Régnier-Vigouroux

**Affiliations:** 1 Molecular Cell Biology, Institute of Zoology, Johannes Gutenberg University of Mainz, Mainz, Germany; 2 CLADIAC GmbH, Heidelberg, Germany

## Abstract

Multicellular tumor spheroids embedded in a matrix represent invaluable tools to analyze cell invasion. Spheroid sizes and invasiveness are the main observables easily measurable to evaluate effects of biological or pharmaceutical manipulations on invasion. They largely account for these 3-D platforms variability, leading to flaws in data interpretation. No method has been established yet that characterizes this variability and guarantees a reliable use of 3-D platforms. Spheroid initial/end sizes and invasiveness were systematically analyzed and compared in spheroids of U87MG cells generated by three different methods and embedded at different times in a collagen matrix. A normality test was used to characterize size distribution. We introduced the linearity-over-yield analysis as a novel mathematical tool to assess end sizes and invasion reproducibility. We further provide a proof of concept by applying these tools to the analysis of a treatment known to be effective beforehand. We demonstrate that implementation of these statistical and mathematical tools warranted a confident quantification and interpretation of in 3-D conducted assays. We propose these tools could be incorporated in a guideline for generation and use of 3-D platforms.

Tissue microenvironments are complex and dynamic systems that provide the prerequisites to disease development and progression^1^. In cancer, microenvironments support signaling from non-tumor cells, e.g. immune cells, which triggers changes in the chemical and physiological conditions. This leads among others to remodeling of the extracellular matrix^2^ (ECM) and to the facilitation of tumor cells migration towards the nearby regions into the ECM^3^. These latter cellular processes constitute relevant targets for cancer therapy and are widely studied *in vitro* using conventional two-dimensional (2-D) cultures as experimental platforms and high-throughput systems for anti-cancer drug therapy testing^4^. Although these systems offer a fast read-out for tumor response to treatment, they have various limitations^5^ when compared with genuine *in vivo* structures. In answer to this issue, the design of novel and complex three-dimensional (3-D) cell culture systems that mimic the *in-vivo* human tissue dynamics, e.g. solid tumors, and their use in cancer research has increased in the recent years^6^. Such systems indeed provide a unique *in vitro* possibility to study in an accurate way behavioral changes in cells and microenvironments. They serve as experimental platforms to investigate molecular signaling in the context of effect and functionality^7^. The challenge is to adapt to these 3-D systems the 2-D cell based assays that facilitate the investigation of cell response to stimuli propagated in the microenvironment^8^ (e.g. signal transduction, gene and protein expression).

*In vitro* micro-scale tissue-like structures, such as multicellular spheroids, are models that provide *in vivo*-resembling in-cell arrangement. Cell arrangement in spheroids made of tumor cells for instance mimic the *in vivo* conditions presented upon tumor occurrence^9^. Profiles of cell distribution, proliferation and survival in spheroids define oxygen or nutrient gradients and pH variations that are similar to that observed in tumors. These profiles dictate the metabolic activity of the cell^10^ which, in cancer cells, directly influences the invasiveness of tumor cells into the ECM^11^,^12^. In the process of cell invasion, tumor cells infiltrate the complex network of proteins that forms the ECM structure^13^. In 3-D cultures, contrary to 2-D cultures, cell structure and polarization are preserved during the proteolytic activity of proteins secreted by invading cells and remodeling of ECM occurs in real time^13^,^14^. The spheroid cell arrangement and the cell invasion mechanisms behave in a particular manner^15^ that depends on the tumor model, as defined by the tumor cell line studied, the method chosen to generate spheroid and the ECM characteristics of the chosen matrix^16^,^17^. In order to obtain accurate and reproducible results within one model and to extend these observations to further models, variations thus need to be identified and quantified. For instance, it is necessary to understand how similarly spheroids generated by different techniques invade in a given matrix. If initial sizes of spheroids and their invasiveness were to be distributed at random, then any experiment conducted with these spheroids would be inconclusive, since the differences in behavior between different groups could not be clearly attributed to a condition. It would be just as likely in this case that the changes are of random nature.

In this study, we intended to quantify the random influence of different spheroid generation methods and its consequences on reproducibility and correctness of results when performing invasion assays. For that purpose, we employed three different methods to generate spheroids of the same cell line, U87MG glioblastoma cells, and embedded them in a collagen I matrix at two different time points following their generation. We systematically analyzed and compared first hand observables, i.e. the uniformity of initial spheroid size, the uniformity of final spheroid size and invasiveness. To assess the effectiveness of our statistical and mathematical tools, we applied them to analyse the data of an invasion assay, in which the effects of a sphingosine kinase inhibitor^18^ on spheroids generated with one of the utilized methods were tested. Our data indicate that information about these observables constitutes the basis for a set of minimal information required to define the variability of a given tumor model. Because this information will determine the conclusiveness of the obtained results, we propose that this set of information shall be provided by researchers as a quality assessment and validation of the model used to conduct *in vitro* invasion assays.

## Results

### Uniformity of initial spheroid sizes

**Figure 1:**
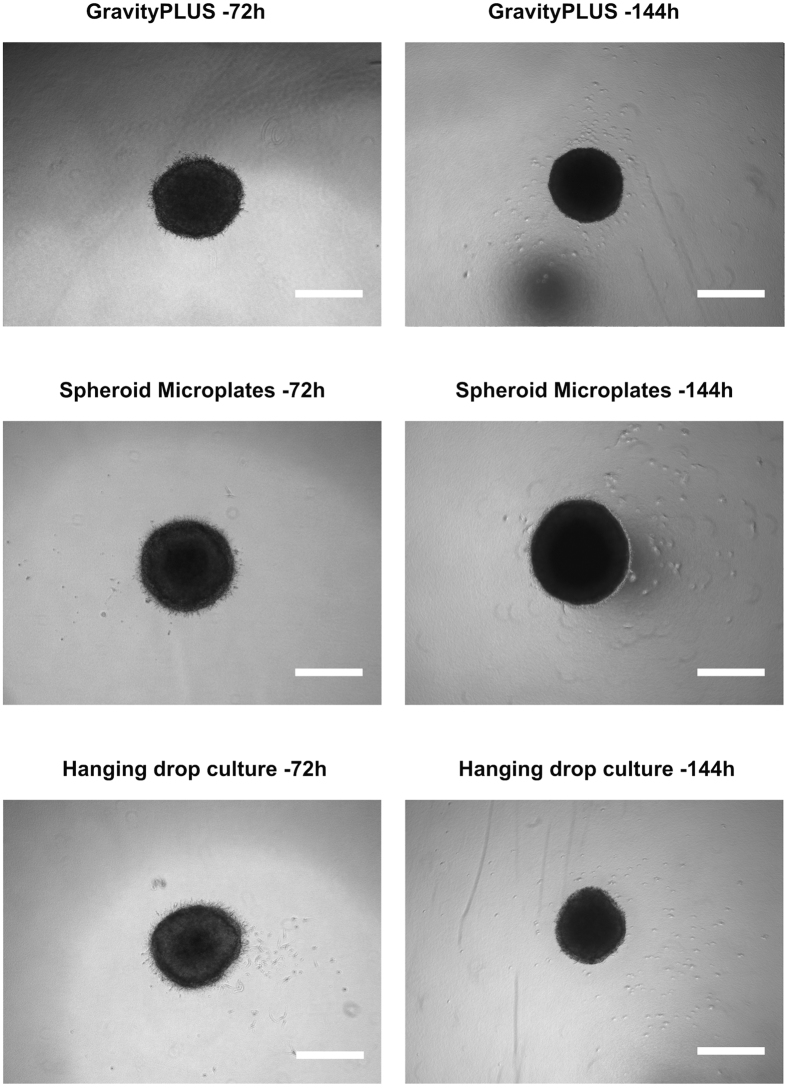
Representative images of U87MG cells spheroids at day 0. Spheroids were generated by the three different methods and embedded after 72 h and 144 h. All images were acquired with an inverted light microscope at 4-fold magnification 3 h after embedding. Scale bar: 500 μm.

**Figure 2:**
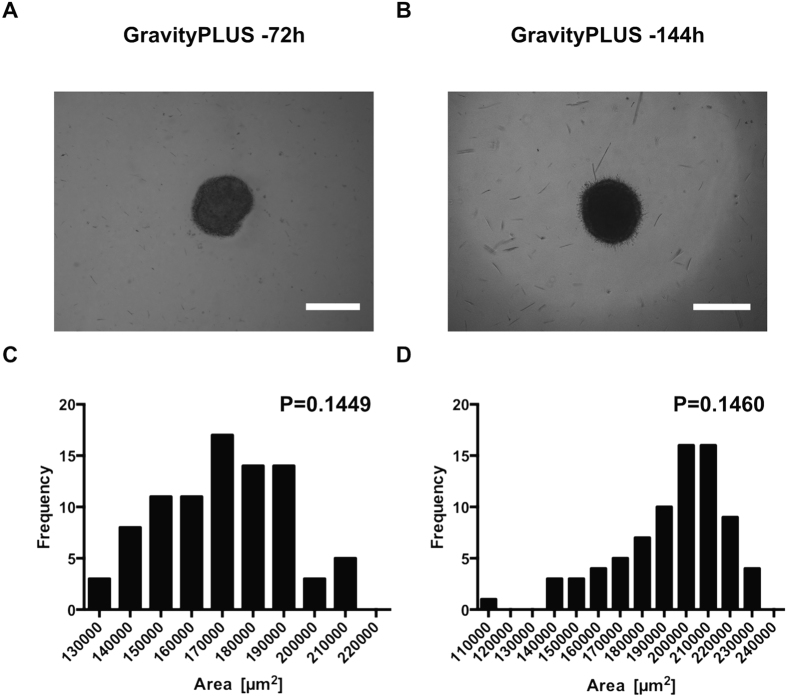
Distribution of initial sizes of spheroids generated by the GravityPLUS hanging drop system. (**A**,**B**) are representative images of U87MG spheroids generated at 72 h and 144 h after embedding (day 0) in the collagen matrix containing sphingosine kinase inhibitor (30 μM final concentration). (**C**,**D**) represent the distribution of initial (day 0) sizes expressed in squared micrometers [μm^2^] at the two different time points 72 h (**C**) and 144 h (**D**) after spheroid generation. P: value derived from the normality test. The test is passed if P > 0.05. n = 3.

Testing the effects of a treatment on cell invasion in the collagen matrix requires that initial conditions of test are comparable. This means that spheroids should present the same or very similar initial size. For each method of spheroid generation, the initial sizes of spheroids are expected to be normally distributed. If the method used is robust and leads to repeatable results, the variances in initial sizes are expected to arise only due to statistical fluctuation. A frequency analysis of initial sizes for the different methods, followed by a D’Agostino & Pearson omnibus normality test, was conducted to substantiate this assumption. A sample of 24 spheroids for each generation method and each time point (all 3 experiments) was analysed 3 h post-embedding as described in Methods (day 0; Fig. 1). These initial sizes were quantified as the size of the area in squared micrometers [μm^2^] that a spheroid occupies in the collagen matrix in the respective micrograph. Analysis of size distribution (Fig. S1 in supplementary material) suggested a Gaussian distribution of the initial sizes of spheroids generated by each method (see e.g. 144 h for the GravityPLUS method and the Spheroid Microplates method; 72 h for the Hanging drop method). This is supported by analysis of these data with the D’Agostino & Pearson omnibus normality test, which shows all methods passed (Table S1 supplementary material). In order to confirm these observations, we performed an analysis on a larger sample of spheroids (90 spheroids for each time point and all 3 experiments) generated by the GravityPLUS method. Analysis of their initial size indicated a clear Gaussian distribution and the normality test was successfully passed (Fig. 2). Altogether, this suggests that each method facilitates the generation of uniformly-sized spheroids, and therefore uniform initial conditions.

In Table 1, the average initial sizes of the spheroids generated by the three methods are presented, along with their distribution around the average size represented by the standard deviation. Analysis of the standard deviation provides a very important information to exclude the influence of statistical variation on the size and thus to identify the most appropriate method for spheroid generation. The smaller this value is, the more similar the spheroids are in size. Comparison of the standard deviation values indicated no gross differences between the methods, with values ranging from 0.11 × 10^5^ to 0.31 × 10^5^. The exception was the standard deviation value of 0.66 × 10^5^ observed for the average size of spheroids generated by the Spheroid Microplates method with embedding at 144 h. This suggests that the results of an invasion assay using spheroids generated by the Spheroid Microplates method might be less reproducible than the results of invasion assays using spheroids generated by the other methods.

Since monitoring cell invasion by photography is limited by the size of the camera aperture, spheroids with a relatively large initial size can only be reliably followed and analyzed for periods of time shorter than those needed for smaller spheroids. Thus, considering the size parameter, the spheroids that are the most suited for an invasion assay are the smallest ones. These are those generated after 144 h by the GravityPLUS method (average size of 1.79 ± 0.11 × 10^5 ^μm^2^) and those generated after 144 h by the hanging drop method (average size of 1.41 ± 0.22 × 10^5 ^μm^2^). The least suited, being the largest ones, are those generated after 72 h and 144 h by the Spheroid Microplates method, with an average size of (3.41 ± 0.22) × 10^5 ^μm^2^ and (2.86 ± 0.66) × 10^5 ^μm^2^ respectively. These larger sizes might result from cell seeding in a large vessel (area and volume) such as those provided by the Spheroid Microplates (200 μl) that might impose less spatial restriction than those vessels provided by the GravityPLUS method (40 μl) and the hanging drop method (25 μl). However, it is worth noting that spheroids generated by the three methods all undergo compaction with time, as indicated by the comparison of their sizes after 72 h and 144 h of growth. Another indicator of the reproducibility of sizes can be extracted from the analysis of standard deviation of each method compared with its relevant average size. Indeed, standard deviations expressed as percent of average size all fall in the range of 6–23%, indicating that approx. 80% of the spheroids have comparable size.

### Uniformity of invasion progression

**Figure 3:**
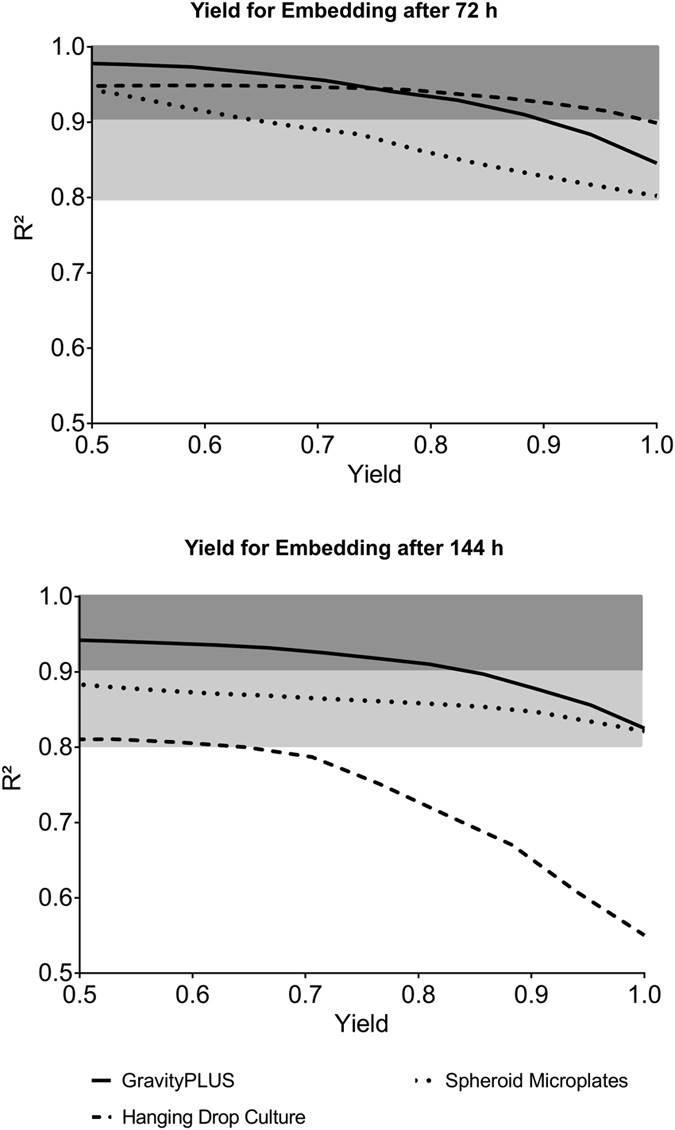
Reproducibility of invasion over 8 days: linearity-over-yield analysis. Yield (ratio of spheroids included in the linear regression analysis) was plotted against linearity (R^2^ value) to characterize the reproducibility of invasion of spheroids generated by the three methods and embedded after 72 h and 144 h. The two grey areas represent a linearity (R^2^) requirement of statistically acceptable strictness. The plot starts at a yield of 0.5, since using less than 50% of generated spheroids is unreasonable. n = 3.

In order to guarantee reproducibility and lower the possibility of false positives and false negatives, it is important that spheroids not only have similar initial sizes, but also invade uniformly under similar boundary conditions. To investigate this issue, the sizes of the spheroids embedded in the collagen matrix (24 for each generation method) were recorded every second day over 8 days. Reproducibility was assessed by comparing all spheroids within one condition (method of generation), using the following approach. Assuming a linear invasion with time, we sought to determine the influence of outliers on the goodness of fit (R^2^). For that purpose, multiple linear regression analyses of invasion data obtained for each condition were performed (Fig. 3), where outliers were gradually excluded while recording R^2^. The yield, i.e. the number of spheroids included in the linear regression analysis was expressed as a ratio (e.g. 1 = all spheroids included; 0.5 = half of the spheroids included) and plotted against R^2^ as a measure of linearity. The plots thus illustrate for the two different embedding time points how linearly (i.e. uniformly) the spheroids from a generation method invade, when outliers are gradually excluded from the assay. The light grey areas in the plots represent a minimal range requirement of R^2^ ≥ 0.80 to R^2^ ≥ 0.90, which is an area where the invasion is sufficiently linear to be considered as uniform. The R^2^ could certainly be set at a value higher than 0.90, but this would result in the exclusion of more and more samples, up to a point where there would be not enough samples left to get reliable statistics. For every method and both embedding time points, the percentage of spheroids that violate the maximum tolerance given by R^2^ is taken out of the fit until the condition is fulfilled. This linearity-over-yield analysis is powerful for determining how uniformly the generated spheroids invade, or, interpreting it the other way around, how many outliers a generation method will produce under the condition of a minimum linearity given by R^2^. A summary of the yields calculated for the different generation platforms and linearity requirements is presented in Table 2. The table provides as an example the most generous minimum requirement of R^2^ = 0.80, and the strictest minimum requirement of R^2^ = 0.90. At the early embedding time point (72 h), the linearity requirement of R^2^ ≥ 0.80 is met by each generation method (ratio of 100% of spheroids); choosing the strictest linearity requirement of R^2^ ≥ 0.90 still leads to a high ratio of spheroids invading consistently: 100% spheroids for the hanging drop culture, 90% for the GravityPLUS method, and 65% for the Spheroid Microplates method. At the later embedding time point (144 h), this strictest linearity requirement of R^2^ ≥ 0.90 is met only by the GravityPLUS method (ratio of 85% spheroids). Reducing the linearity requirement to R^2^ ≥ 0.80 ensures invasion reproducibility of 100% spheroids generated by both the GravityPLUS and Spheroid Microplates methods, but only of 65% spheroids generated by the hanging drop method.

**Figure 4:**
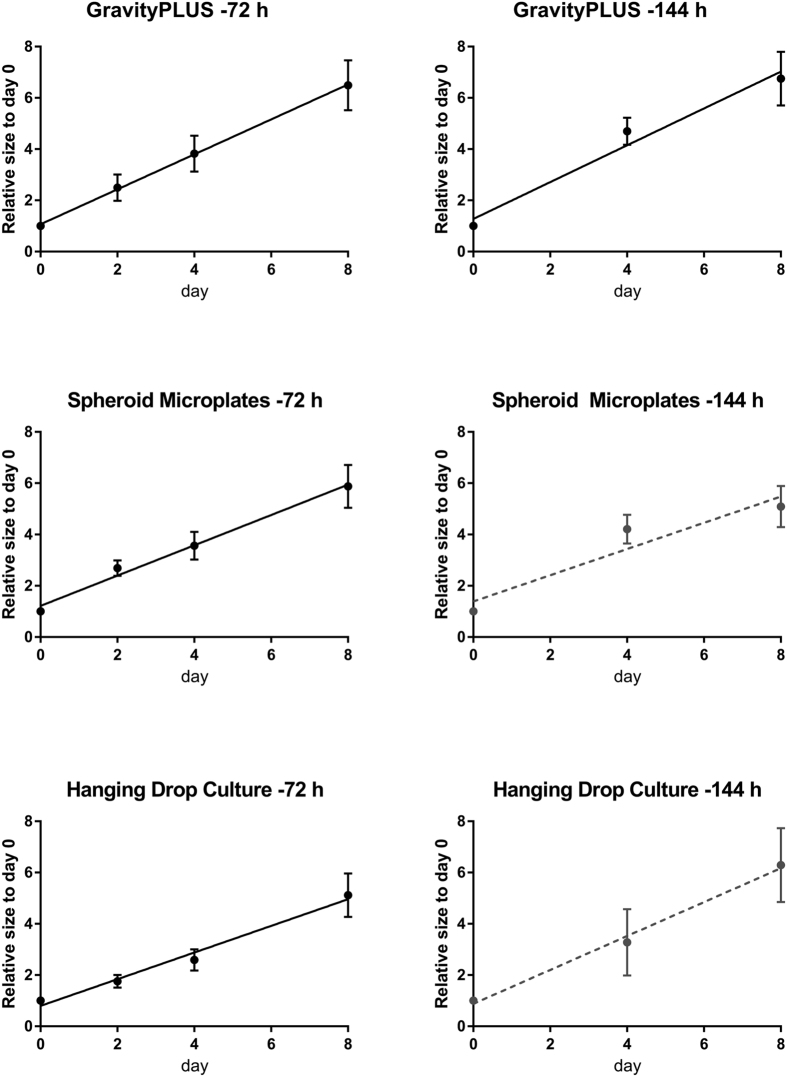
Progression of invasion of spheroids generated by the three methods. Average end sizes expressed as relative size to day 0 were plotted against time. The full lines result from linear regression analyses that exclude outliers above R^2^ = 0.90. The dashed lines show linear regression analyses with R^2^ above 0.80 (Spheroid Microplates and hanging drop methods, embedding after 144 h).

The invasion data of spheroids selected according to the linearity-over-yield analysis is presented in Fig. 4, with the average relative size for each embedding time point plotted with its standard deviation as error bars against time. Table 3 presents the fit parameters for the linear regression performed for every method. Of the three generation methods, the GravityPLUS method provides the fastest invading spheroids with a slope of (0.68 ± 0.03) pdu/day for embedding after 72 h and (0.71 ± 0.04) pdu/day for embedding after 144 h, whereas spheroids generated by the Spheroid Microplates method show the lowest invasion with (0.51 ± 0.03) pdu/day for embedding after 144 h.

### Uniformity of final size

**Figure 5:**
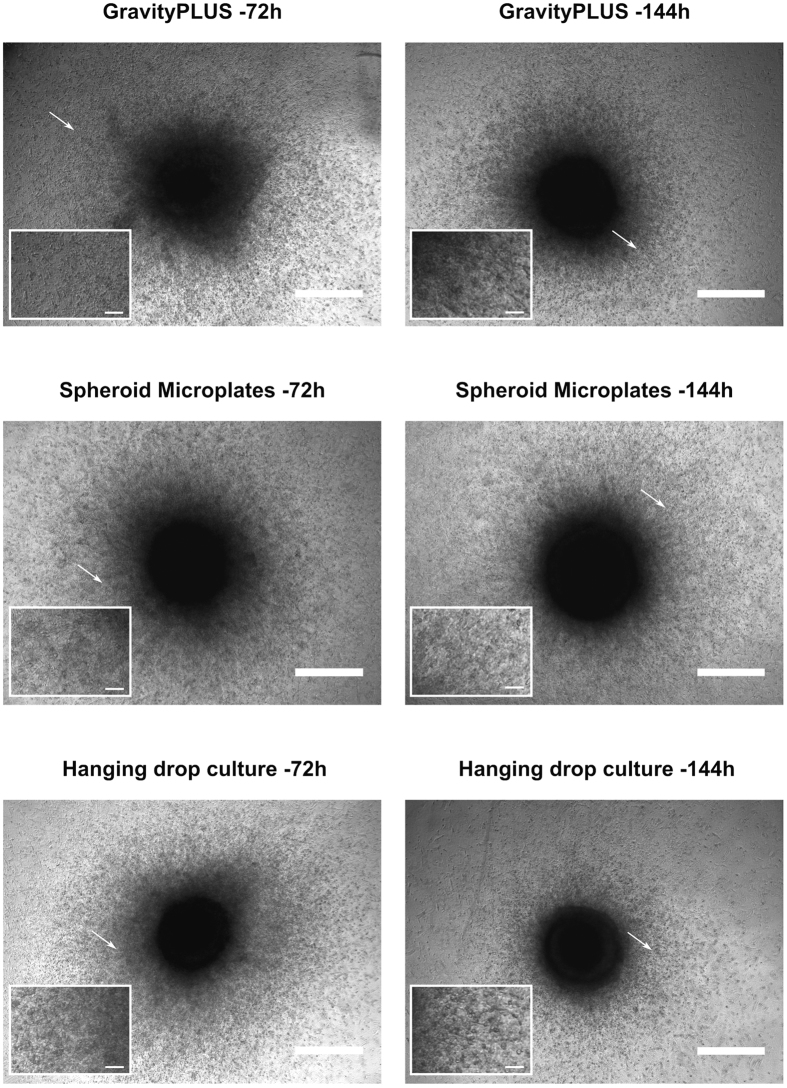
Invasion of U87MG cells spheroids generated by the three methods and embedded after 72 and 144 h of generation. Representative images of U87MG spheroids after 8 days of culture in the collagen matrix. All images were acquired with an inverted light microscope at 4-fold magnification. Scale bar: 500 μm. Migrating cells are depicted in the inserts (40-fold magnification of the region indicated by the white arrow). Scale bar: 100 μm.

**Figure 6:**
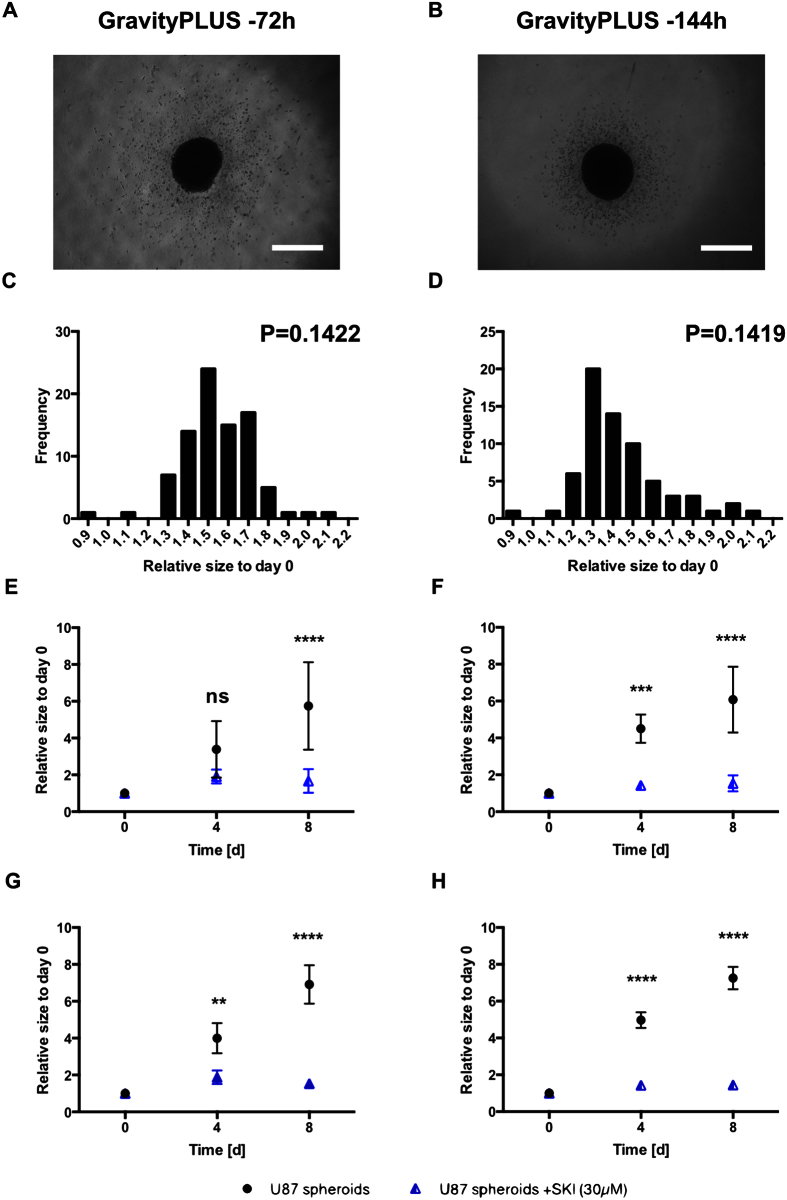
Invasion of SKI-treated U87MG cells spheroids generated by the GravityPLUS hanging drop system and embedded after 72 and 144 h of generation. (**A**,**B**) are representative images of U87MG spheroids generated at 72 h and 144 h after 8 days of culture in the collagen matrix containing SKI (30 μM final concentration). All images were acquired with an inverted light microscope at 4-fold magnification. Scale bar: 500 μm. (**C,D**) represent the distribution of the end sizes (day 8) in relative units of spheroids generated and embedded at the two different time points, 72 h (**C**) and 144 h (**D**). P: value derived from the normality test. The test is passed if P > 0.05. (**E,F**) represent the quantification of invasion of the treated and untreated U87MG spheroids after 72 h (**E**) and 144 h (**F**) of generation before exclusion of the outliers. (**G,H**) represent the quantification of invasiveness of the treated and untreated U87MG spheroids after 72 h (**G**) and 144 h (**H**) of generation after exclusion of the outliers. Data is expressed as mean ± standard deviation of the relative size of the spheroids of the indicated time. **p < 0.01; ***p < 0.001; ****p < 0.0001. n = 3.

A common method to analyze the impact of a treatment on cell invasion is to compare the end sizes of spheroids treated in different conditions. After having established the reproducibility of the initial sizes and invasion uniformity, it is necessary to establish the reproducibility of the end sizes. For that purpose, end sizes of spheroids (Fig. 5) were analyzed as described for the initial sizes. Note that end sizes are expressed as normalized data and are given in relative size to the initial (day 0) size. The distribution of the end sizes of spheroids generated by the three methods is represented in Fig. S2 in the supplementary material. Analysis of end size distribution suggested a normal Gaussian distribution of spheroids generated by each method (see e.g. 72 h for the GravityPLUS method; 144 h for the Spheroid Microplates method; 72 h for the hanging drop method). Analysis of the data with the D’Agostino & Pearson omnibus normality test indicating that all methods passed (Table S2 supplementary material). Analysis of a larger sample of spheroids at day 8 confirmed the normality of size distribution (see below, section D and Fig. 6). Tables 1 and 4 provide the corresponding average start and end sizes and their standard deviations. Analysis of the average end sizes shows that the largest end sizes are observed for spheroids generated by the GravityPLUS method, followed by the hanging drop method and finally by the Spheroid Microplates method. This later method generates the largest initial spheroids (see Table 1 and summary in Table S3) that consequently display the smallest increase in size, reflecting the smallest growth and invasion. Spheroids generated by this method appear therefore the least suited to assess the effects of treatment on spheroid growth and invasion. Analysis of the standard deviations shows that the lowest variation is observed for spheroids generated by the Spheroid Microplates method and by the hanging drop method when spheroids are embedded after 72 h (0.43 and 0.44 standard deviation, respectively; Table S3). A standard deviation of 1.04, which represents a non-negligible variation of size, is observed for the spheroids generated by the GravityPLUS method and embedded after 72 h. The same holds true for spheroids generated by the hanging drop culture and embedded after 144 h, with a standard deviation of 1.12.

Altogether, these data suggest that the spheroids best suited for evaluating the effect of a treatment on a long term (here 8 days) would be spheroids generated by the GravityPLUS method and embedded after 144 h of generation, or the spheroids generated by the hanging drop method and embedded after 72 h of generation.

### Proof of concept

In order to test the validity of our tools, we conducted an invasion assay with a larger set of spheroids generated with the GravityPLUS method. We compared the effects of applying a sphingosine kinase inhibitor (SKI), known to impair survival and invasion of the glioma cells^18^, on spheroids generated after 72 h (87 spheroids of 90) and 144 h (78 spheroids of 90) (Fig. 6). Analysis of the spheroids indicated a normal size distribution confirmed by the normality test at day 0 (see Fig. 2C,D) and at day 8 (Fig. 6C,D). Application of the linearity-over-yield tool to the data with R^2^ ≥ 0.90 indicated 10% of outliers at 72 h and 20% at 144 h. The results of the invasion assay before and after exclusion are shown in Fig. 6. As indicated by the kinetics of invasion depicted in Fig. 6E–H, there was a clear effect of the treatment on the spheroids compared to the controls. Exclusion of the outliers (Fig. 6G,H) clearly impacted on the significance of the results, as most evidently shown at day 4 (compare Fig. 6E–G and Fig. 6F–H).

## Discussion

In this study, we present the analysis of simple observables with a set of statistical and mathematical tools to quantify the variability that spheroid generation techniques impose on invasion assays. We applied these tools to different generation methods to illustrate their usage and justify the necessity of this analysis. The resulting data are reported in Table S3. As a summary of our observations, we propose the following flow that could be used by the researcher to ascertain the accuracy of the 3-D spheroid invasion assay that she/he uses for a given cell line:

1. Analyse the distribution of initial and end sizes: use the normality test and analysis of the standard deviation values for initial and end sizes to pre-select the best conditions (time of embedding, amount of cells…).
2. Analyse the kinetics of invasion with multi-linear regression analyses to identify and exclude outliers; define yield and linearity (R^2^) and plot them to assess consistency of invasion and set the tolerance threshold.

These analyses are discussed in the following sections.

Besides the obvious relevance of initial and end sizes to determine the kinetics of invasion of spheroids, these two observables are crucial to assess the quality of the method chosen to generate spheroids. Both give direct access to the robustness of the method through the evaluation of the reproducibility of spheroid size. At the time of spheroid embedding in the matrix, the measurement of initial sizes gives an essential information about size distribution, whose normality can be tested, and about the behavior of spheroids generated by the chosen method. We observed that, whatever the method used, spheroids after 144 h of generation are more compact (smaller average size compared to those generated after 72 h), a condition which bears a practical advantage (allows for invasion assay over a longer period of time) and a biological one (formation of a less oxygenated core). At the end of the invasion assay, analysis of the U87MG spheroids shows a size distribution that reflects the initial distribution, supporting the linearity of growth/migration of cells into the matrix. If selection of a method were to be based on initial and end sizes of the U87MG spheroids, the Spheroid Microplates method with an incubation time of 72 h would appear the best suited as it offers the highest reproducibility in size (lowest standard deviations). However, these spheroids are the largest at the beginning of the assay, a condition that dramatically reduces the physical space available for invasion and hence decreases its experimental potential for drug testing. Indeed, to serve as experimental platforms for anti-cancer drug testing, spheroids must facilitate a clear detection of the impact of a drug/treatment on cell survival/invasion over the experimental time period (e.g. 8 days in the case of our study). In that regard, the method of choice appears to be the GravityPLUS method, with a generation time of 144 h (normal distribution, low standard deviation). The method allows for the largest end size, i.e. the highest fold of growth (=higher slope), which represents an ideal condition for testing drugs with (early) effects on growth and/or invasion in the frame of the experimental 8 days set-up.

Analysis of the kinetics of invasion after drug treatment provides essential information about the timing (when the drug starts to affect the cells) and strength of the drug effect (change in the slope of the growth/invasion curves). These changes must be specific and not random. Thus, the prerequisite is a robust, reproducible experimental system. Analysis of the kinetics of U87MG spheroids invasion led us to develop and implement a novel mathematical tool for quantifying the reproducibility of invasion: the linearity-over-yield analysis. This analysis proved to be very helpful to define consistency in our culture systems.

For instance, in the case of an invasion assay conducted with spheroids generated by the GravityPLUS method, we observed a ratio of consistent invasion of 90% for a good linearity requirement of R^2^ ≥ 0.90. This means that 10% of spheroids are expected to behave differently, not because of any treatment, but simply because they belong to the allowed percentage of not uniformly invading samples and as such must be disregarded. This is especially dramatic in the case of the hanging drop culture method (with embedding after 144 h) and the Spheroid Microplates platform (with embedding after 72 h). With a reproducibility of 65%, it means that 35% of the samples will not show consistent behavior, making an analysis of the impact of a treatment on the invasion difficult. If these generation methods have to be used by the experimenter, one way to counter-act this effect would be to run a large number of replicates, in order to achieve a number of samples high enough for statistical significance after having discarded 35% of the samples. This, however could lead to an easy justification for disregarding samples and therefore to the issue of “cherry-picking” results. We thus favor the following approach: adjust the percentage to the minimal value needed to observe an effect; once this minimal percentage of reproducibility has been chosen, it must be raised according to the expected variability linked to the generation method. As an example, the minimum of differently behaving samples could be set to 50% to be able to speak of a first indicator of an effect. For the GravityPLUS method with embedding after 72 h, this would translate into the condition that a minimum of 60% of spheroids must behave differently in order to account for the 10% that would have invaded differently anyway. For the hanging drop culture method with embedding after 144 h, the same approach would result in the condition that 50% + 35% = 85% of the samples must show a consistent different behavior.

Another factor influences the statistical evaluation of an invasion assay and this is the tolerance in invasion uniformity, here given as the linearity requirement value R^2^ ≥ 0.90 from the multilinear regression analyses of invasion. The yield of spheroids that show a reproducible invasion varies with the value set for the linearity requirement, i.e. for the tolerance. When comparing two conditions of treatment for instance, it will thus be very important to ensure that the linearity-over-yield analysis for each set of data indicates reproducibility of invasion in each condition (e.g. control versus treatment) with the same linearity requirement value R^2^ ≥ 0.90. Lowering the tolerance threshold to R^2^ = 0.80 will result in including more samples in the regression analysis which could help raising the yield, but could also result in masking differences in the conditions tested. We therefore favor the use of stringent values for setting the tolerance. As seen in Fig. 6, stringent values led to a much clearer difference in the kinetics of the proof of concept experiment, facilitating the distinction between conditions. Furthermore, the respective kinetics represent three independent experiments, and the exclusion of outliers according to the linearity-over-yield for the utilized generation method drastically reduced the uncertainty of the kinetics. As a consequence, the reproducibility of the three independent experiments is much more evident.

Each method has its pros and cons. One method can facilitate a higher yield of reproducible uniformly invading spheroids that however show variability in their end sizes (such as the spheroids generated in our study with the hanging drop culture method). Or it can facilitate the generation of compact spheroids but at the cost of the yield, requiring thus to run more samples for achieving statistical significance, or to define a stringent minimum ratio of differently behaving spheroids (such as the spheroids generated with the Spheroid Microplates method). Thus, it is clear that a trade-off needs to be made by the researcher to select the most appropriate method according to the working objectives. Based on our observations, we thus would recommend that the method used in a laboratory to generate spheroids for invasion assays be tested for its accuracy according to the flow we proposed at the beginning of the discussion. We noted as well in the course of this study the relevance of the time point of generation for the assay consistency and would recommend to test this parameter as well. In summary, we believe that the analyses we propose to perform will be of great help to assist researchers because they provide them with tools to assess the statistical variations and hence reproducibility of their experimental spheroid system, a necessary basis to warrant an unbiased interpretation of data. To the best of our knowledge, this is the first work that introduces such tools for quality assessment of spheroid generation methods. We hope these tools will facilitate a standardization of 3-D cultures, guaranteeing their reliability and promoting their broader use.

## Methods

### Cell culture

U87MG human glioblastoma cell line was purchased from the American Type Culture Collection (ATCC). Cells were cultured in high-glucose Dulbecco’s Modified Eagle’s Medium (DMEM) and supplemented with 10% heat-inactivated fetal calf serum (FCS), 1% L-glutamine (2 mM) and 1% gentamicin (50 units per ml), all from Sigma-Aldrich. This medium is referred to as complete DMEM (cDMEM). The FCS concentration was reduced to 5% in medium used for experiments (cDMEM-5). Cells were maintained in standard culture conditions (37 °C in humidified air with 5% CO_2_).

### Generation of multicellular spheroids

#### Manual hanging drop technique

U87MG cells expanded in cell-culture flask were detached with 0.25% trypsin/EDTA (Invitrogen) and re-suspended with cDMEM at the final concentration of 10^6^ cells/ml. 25 μl of this cell suspension (i.e. 25000 cells) were deposited in a drop-like form on the lid of a 10 cm Petri dish which was flipped back to the dish containing 8 ml of phosphate-buffered saline (PBS). Drops were incubated at standard culture conditions for 72 h and 144 h.

#### Gravity PLUS hanging drop system

U87MG cells prepared as described above were resuspended in cDMEM at the final concentration of 25000 cells in 40 μl. Cells were distributed (40 μl/well) in the GravityPLUS 96-well plate according to the manufacturer’s instructions (InSphero). Plates were incubated at standard culture conditions for 72 h and 144 h.

#### Spheroid Microplates

U87MG cells were resupended in cDMEM at the final concentration of 25000 cells in 200 μl. Cells (200 μl per well) were distributed in flat 96-well low attachment surface plates (Corning). Plates were incubated at standard culture conditions for 72 h and 144 h.

For each method, three independent experiments (96 spheroids generated) were conducted.

### 3-D invasion assay

After 72 h and 144 h of culture, U87MG cell spheroids were implanted in the center of each well of a 24-well plate coated with a 2.2 mg/ml collagen mixture (one spheroid per well in 400 μl of collagen mixture per well). For each of the three independent experiments, 8 spheroids generated by each of the three methods were randomly chosen for embedding. The collagen mixture was prepared by mixing 2 ml of PureCol bovine collagen type I solution (3 mg/ml; Advanced BioMatrix) with 250 μl of 10X minimal essential medium (MEM) (Sigma-Aldrich) and 500 μl of sodium hydroxide 0.1 M. After cell spheroid embedding, the plate was incubated for 20 min at standard culture conditions to solidify the gels. Thereafter 400 μl of cDMEM was overlaid on the collagen matrix in each well. The complete system was incubated for a total of 8 days.

### Sphingosine Kinase Inhibitor (SKI) treatment on multicellular spheroids

After 72 h and 144 h of culture, U87MG cell spheroids generated by the GravityPLUS hanging drop system were implanted in the center of each well of a 24-well plate coated with a 2.2 mg/ml collagen mixture (one spheroid per well in 400 μl of collagen mixture per well). For each of the three independent experiments, 30 spheroids were randomly chosen for embedding. The sphingosine kinase inhibitor (Sigma) was added at a final concentration of 30 μM to the liquid collagen mixture (prepared as described in C). After cell spheroid embedding, the plate was incubated for 20 min at standard culture conditions to solidify the gels. Thereafter 400 μl of cDMEM was overlaid on the collagen matrix in each well. The complete system was incubated for a total of 8 days.

### Quantification of spheroid size and invaded area

After the spheroids were embedded, cell invasion out of the spheroid was monitored by digital photography using a Leica DM IL LED Fluo inverted light microscope (Leica DFC450C camera) at room temperature, with the Leica Application Suite (LAS V4.4). Images were acquired every second day (day 0 = time of embedding in collagen; picture taken 3 h after embedding) using a 4x/0.10 objective. Processing of raw images and quantification of spheroids and of invasion areas was performed using an in-house software. This in-house software takes into account cell density and not the (observer-dependent) limits of cell migration in the collagen matrix. The implemented algorithm uses local fluctuations of the image intensity for an automated estimation of the invasion magnitude. It is robust enough to handle micrographs of different generation methods and various qualities without the concept of an invasive front of the spheroids (Cisneros *et al*. 2016, manuscript submitted for review). Sizes at day 0 (after 72 h and 144 h of spheroids generation) are expressed in squared micrometers (μm^2^), which were converted from the acquired squared pixels (px^2^) using a conversion factor. The factor was calculated by dividing the 500 μm scale bar (provided by LAS V4.4) by the length in pixels that the scale bar occupies in a micrograph, and therefore is a measure how many micrometers fit in one pixel. Invasion and spheroid areas are normalized for each day to the invasion and spheroid areas measured at day 0 and expressed in protocol defined units (PDU). These normalized data are reported as relative size to day zero. Relative size of day 0 thus equals 1.

Quantification was performed with a maximum of 24 samples (8 spheroids/experiment, three independent experiments). We excluded from the quantification images of spheroids that displayed post-embedding (thus unrelated to the generation method) abnormalities in their location in the matrix (drift form the center to the edge of the well) or in the matrix morphology (loss of structure).

In the case of the invasion assay conducted with a large sample of spheroids (90) generated with the GravityPLUS system, 87 spheroids (after 72 h of generation) and 78 spheroids (after 144 h of generation) out of the 90 samples did not show post-embedding abnormalities and were used for analysis.

Representative images shown in the figures were enhanced in brightness and contrast using GNU Image Manipulation program (GIMP), which is an open-source raster graphics editor. All images were increased in brightness and contrast to a value of 60 and 40 respectively. Inkscape software, which is an open-source vector graphic editor, was used to generate the close-up (40-fold magnification) sections of the images in Fig. 5.

### Statistical Analysis

All statistical analyses were performed using Prism (GraphPad). The distribution of initial spheroid sizes was analyzed with the D’Agostino & Pearson omnibus normality test. Linear regression and comparison of the data was performed with data from three independent experiments. Non-linear Gaussian regression of the data was performed with data from three independent experiments. Statistical significance of data obtained from at least 3 independent experiments was determined using two-way ANOVA analysis of variance (ANOVA) combined with Bonferroni analysis with P < 0.05 being considered statistically significant. Data are expressed as mean ± standard deviation (SD) of at least 3 independent experiments.

A novel method for the quantification of the factor [reproducibly invading spheroids per experiment] that we named linearity-over-yield (see Results, section B) was implemented in MATLAB (the code can be made available upon request).

## Additional Information

**How to cite this article**: Cisneros Castillo, L. R. *et al*. Evaluation of Consistency in Spheroid Invasion Assays. *Sci. Rep.* **6**, 28375; doi: 10.1038/srep28375 (2016).

## Supplementary Material

Supplementary Information

## Figures and Tables

**Table 1:** Average initial sizes of spheroids generated by the three methods.

Method	μ [10^5^ μm^2^]	σ [10^5^ μm^2^]
GravityPLUS-72 h	2.17	0.31
GravityPLUS-144 h	1.79	0.11
Spheroid Microplates-72 h	3.41	0.22
Spheroid Microplates-144 h	2.86	0.66
Hanging drop culture-72 h	2.35	0.22
Hanging drop culture-144 h	1.41	0.22

Data represents the average initial sizes (μ) expressed as squared micrometers and standard deviations (σ) of the spheroids analyzed in Fig. S1 in supplementary material (day 0). n = 3.

**Table 2:** Yields of reproducibly invading spheroids.

Embedding Method	72 h		144 h	
	R^2^ ≥ 0.80	R^2^ ≥ 0.90	R^2^ ≥ 0.80	R^2^ ≥ 0.90
GravityPLUS	100%	90%	100%	85%
Spheroid Microplates	100%	65%	100%	–
Hanging drop culture	100%	100%	65%	–

Yields, expressed as ratio (%), of consistently invading spheroids generated by the three methods and embedded after 72 h and 144 h are presented for each minimal linearity requirement R^2^ = 0.80 and R^2^ = 0.90. The higher the ratios fitting within the two minimum linearity requirements, the higher the reproducibility. n = 3.

**Table 3:** Fit parameters of the linear regression analysis for the invasion assays.

Method	Slope [pdu/day]	Intercept [pdu]	R^2^
GravityPLUS-72 h	0.68 ± 0.03	1.07 ± 0.12	0.91
GravityPLUS-144 h	0.71 ± 0.04	1.27 ± 0.19	0.91
Spheroid Microplates-72 h	0.59 ± 0.03	1.21 ± 0.13	0.91
Spheroid Microplates-144 h	0.51 ± 0.03	1.39 + 0.16	0.82
Hanging drop culture-72 h	0.52 ± 0.01	0.79 ± 0.07	0.90
Hanging drop culture-144 h	0.66 ± 0.06	0.88 ± 0.30	0.80

Data represents the resulting slope, intercept and goodness of fit (R^2^) of the regression lines determined for each method at both embedding times. n = 3.

**Table 4:** Average end sizes of spheroids after 8 days of invasion.

Metho	μ [size(day 0)]	σ [size(day 0)]
GravityPLUS-72 h	6.91	1.04
GravityPLUS-144 h	7.30	0.61
Spheroid Microplates-72 h	4.68	0.43
Spheroid Microplates-144 h	5.00	0.52
Hanging drop culture-72 h	5.39	0.44
Hanging drop culture-144 h	6.48	1.12

Spheroids were generated by the three different methods and embedded after 72 h and 144 h. Sizes (μ) and standard deviations (σ) are expressed as relative sizes (size relative to day 0) at day 8. n = 3.
