# Supplementary material for: Evaluation of Consistency in Spheroid Invasion Assays
Source: Sci Rep. 2016 Jun 23;6:28375. doi: 10.1038/srep28375 (PMC4917829; doi:10.1038/srep28375)
Supplement: Supplementary Information [file srep28375-s1.pdf]

Supplementary Material

Evaluation of Consistency in Spheroid Invasion Assays

Liliana R. Cisneros Castillo[1], Andrei-Dumitru Oancea[2], Christian Stüllein[2], and Anne Régnier-Vigouroux[1],*

[1]Molecular Cell Biology, Institute of Zoology, Johannes Gutenberg University of Mainz, Mainz, Germany

[2]CLADIAC GmbH, Heidelberg, Germany

*Corresponding Author:

Anne Régnier-Vigouroux

Johannes Gutenberg University of Mainz,

Johann-Joachim-Becher-Weg 15

55128 Mainz, Germany

Tel.: 0049-6131-39 23 949

E-mail address: vigouroux@uni-mainz.de

Figure S1

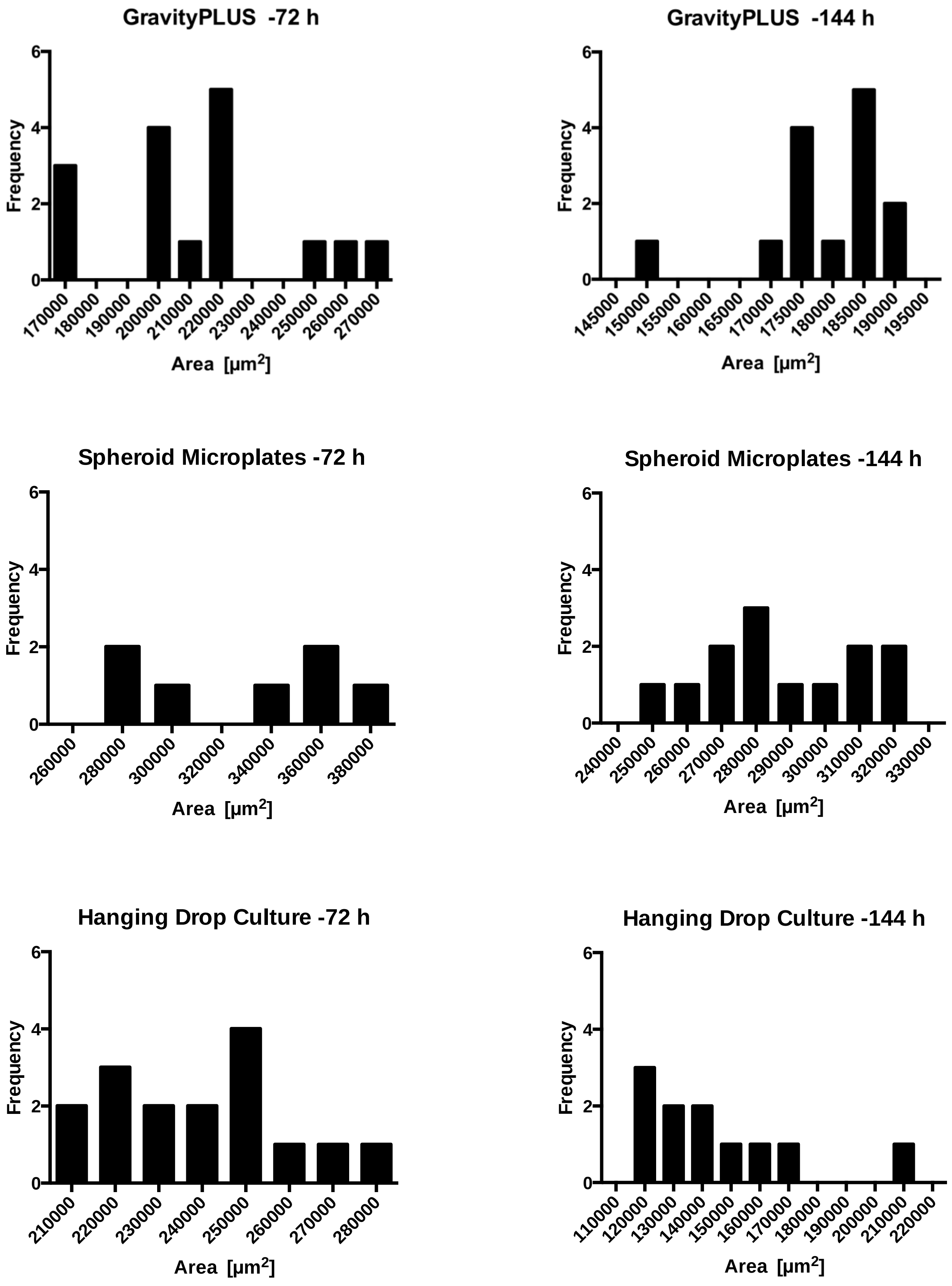
GravityPLUS -72 h
Frequency
0
2
4
6
170000
180000
190000
200000
210000
220000
230000
240000
250000
260000
270000
Area [µm2]
GravityPLUS -144 h
Frequency
0
2
4
6
145000
150000
155000
160000
165000
170000
175000
180000
185000
190000
195000
Area [µm2]
Spheroid Microplates -72 h
Frequency
0
2
4
6
260000
280000
300000
320000
340000
360000
380000
Area [µm2]
Spheroid Microplates -144 h
Frequency
0
2
4
6
240000
250000
260000
270000
280000
290000
300000
310000
320000
330000
Area [µm2]
Hanging Drop Culture -72 h
Frequency
0
2
4
6
210000
220000
230000
240000
250000
260000
270000
280000
Area [µm2]
Hanging Drop Culture -144 h
Frequency
0
2
4
6
110000
120000
130000
140000
150000
160000
170000
180000
190000
200000
210000
220000
Area [µm2]

Figure S2

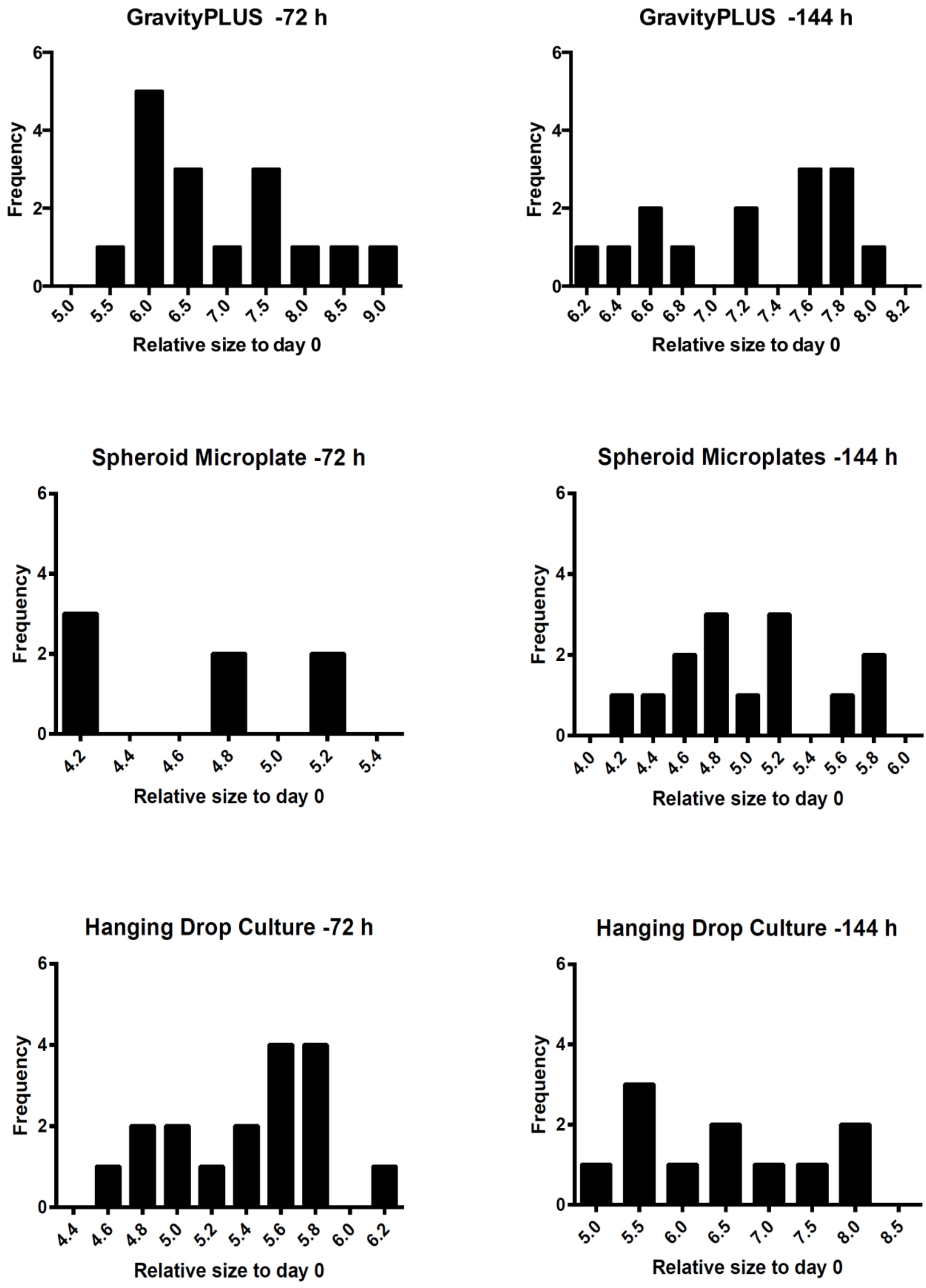

GravityPLUS -72 h
Frequency
0
2
4
6
5.0
5.5
6.0
6.5
7.0
7.5
8.0
8.5
9.0
Relative size to day 0
GravityPLUS -144 h
Frequency
6.2
6.4
6.6
6.8
7.0
7.2
7.4
7.6
7.8
8.0
8.2
Relative size to day 0
Spheroid Microplate -72 h
Frequency
4.2
4.4
4.6
4.8
5.0
5.2
5.4
Relative size to day 0
Spheroid Microplates -144 h
Frequency
4.0
4.2
4.4
4.6
4.8
5.0
5.2
5.4
5.6
5.8
6.0
Relative size to day 0
Hanging Drop Culture -72 h
Frequency
4.4
4.6
4.8
5.0
5.2
5.4
5.6
5.8
6.0
6.2
Relative size to day 0
Hanging Drop Culture -144 h
Frequency
5.0
5.5
6.0
6.5
7.0
7.5
8.0
8.5
Relative size to day 0

Figure Legends

Figure S1: Distribution of initial sizes of spheroids generated by the three different methods. Data represent the prevalence of initial sizes expressed in squared micrometers [μm^2] for the three generation methods at the two embedding time points after 72 h and 144 h. Representative images of the spheroids are shown in Figure 1. n = 3

Figure S2: Distribution of end sizes of spheroids after 8 days of invasion. Data represent the frequency of final sizes in relative units of spheroids generated by the three different methods and embedded after 72 h and 144 h. Representative images of the spheroids are shown in Figure 5. n = 3.

TABLE S1: Distribution of initial sizes of spheroids generated by the three different methods: Normality test. The D'Agostino & Pearson omnibus normality test was performed on sizes of spheroids generated by the GravityPLUS, Spheroid Microplates and hanging drop methods and embedded in collagen at the generation time points of 72 h and 144 h (day 0). Data represent the reported P-Values obtained from a total of three independent experiments (n = 3). The test is passed if P > 0.05.

Method	Passed	P-Value
GravityPLUS-72 h	yes	0.35
GravityPLUS-144 h	yes	0.46
Spheroid Microplates-72 h	yes	0.65
Spheroid Microplates-144 h	yes	0.84
Hanging drop culture-72 h	yes	0.93
Hanging drop culture-144 h	yes	0.44

Table S2: Distribution of final sizes of spheroids generated by the three different methods: Normality test. The D'Agostino & Pearson omnibus normality test was performed on end sizes (day 8) of spheroids generated by the GravityPLUS, Spheroid Microplates and hanging drop methods and embedded in collagen at the generation time points of 72 h and 144 h. Data represent the reported P-Values obtained from a total of three independent experiments. The test is passed if P > 0.05.

Method	Passed	P-Value
GravityPLUS-72 h	yes	0.17
GravityPLUS-144 h	yes	0.61
Spheroid Microplates-72 h	yes	0.19
Spheroid Microplates-144 h	yes	0.61
Hanging drop culture-72 h	yes	0.63
Hanging drop culture-144 h	yes	0.73

Table S3 = Summary of results

Method	Normality test initial/end sizes	Initial size [pdu]	Initial size σ	End size [pdu]	End size σ	Invasion (%) R^2 ≥0.80	Invasion (%) R^2 ≥0.90	Invasion slope
GravityPLUS 72 h	Passed	1.97	2.87	6.91	1,04	100	90	0,68
GravityPLUS 144 h	Passed	1.63	1.10	7.30	0.61	100	85	0.71
Spheroid Microplates 72 h	Passed	3.10	1.97	4.68	0.43	100	65	0.59
Spheroid Microplates 144 h	Passed	2.60	2.60	5.00	0.52	100	-	0.51
Hanging drop culture 72 h	Passed	2.14	1.98	5.39	0.44	100	100	0.52
Hanging drop culture 144 h	Passed	1.28	2.03	6.48	1.12	65	-	0.66
